# Reproducibility and Validity of the 6-Minute Walk Test Using the Gait Real-Time Analysis Interactive Lab in Patients with COPD and Healthy Elderly

**DOI:** 10.1371/journal.pone.0162444

**Published:** 2016-09-08

**Authors:** Wai-Yan Liu, Kenneth Meijer, Jeannet M. Delbressine, Paul J. Willems, Frits M. E. Franssen, Emiel F. M. Wouters, Martijn A. Spruit

**Affiliations:** 1 Department of Research and Education, CIRO, Horn, the Netherlands; 2 NUTRIM School of Nutrition and Translational Research in Metabolism, Maastricht University Medical Centre+, Department of Human Movement Science, Maastricht, the Netherlands; 3 Department of Respiratory Medicine, Maastricht University Medical Centre+, Maastricht, the Netherlands; Universite de Bretagne Occidentale, FRANCE

## Abstract

**Background:**

The 6-minute walk test (6MWT) in a regular hallway is commonly used to assess functional exercise capacity in patients with chronic obstructive pulmonary disease (COPD). However, treadmill walking might provide additional advantages over overground walking, especially if virtual reality and self-paced treadmill walking are combined. Therefore, this study aimed to assess the reproducibility and validity of the 6MWT using the Gait Real-time Analysis Interactive Lab (GRAIL) in patients with COPD and healthy elderly.

**Methodology/Results:**

Sixty-one patients with COPD and 48 healthy elderly performed two 6MWTs on the GRAIL. Patients performed two overground 6MWTs and healthy elderly performed one overground test. Differences between consecutive 6MWTs and the test conditions (GRAIL vs. overground) were analysed. Patients walked further in the second overground test (24.8 m, 95% CI 15.2–34.4 m, p<0.001) and in the second GRAIL test (26.8 m, 95% CI 13.9–39.6 m). Healthy elderly improved their second GRAIL test (49.6 m, 95% CI 37.0–62.3 m). The GRAIL 6MWT was reproducible (intra-class coefficients = 0.65–0.80). The best GRAIL 6-minute walk distance (6MWD) in patients was shorter than the best overground 6MWD (-27.3 ± 49.1 m, p<0.001). Healthy elderly walked further on the GRAIL than in the overground condition (23.6 ± 41.4 m, p<0.001). Validity of the GRAIL 6MWT was assessed and intra-class coefficient values ranging from 0.74–0.77 were found.

**Conclusion:**

The GRAIL is a promising system to assess the 6MWD in patients with COPD and healthy elderly. The GRAIL 6MWD seems to be more comparable to the 6MWDs assessed overground than previous studies on treadmills have reported. Furthermore, good construct validity and reproducibility were established in assessing the 6MWD using the GRAIL in patients with COPD and healthy elderly.

## Introduction

Chronic obstructive pulmonary disease (COPD) is a highly prevalent chronic disease affecting about 10% of adults above the age of 40 [1]. COPD affects respiratory function of patients and has systemic consequences as well, including peripheral muscle dysfunction and weakness, which contributes to exercise limitation and impaired quality of life [2, 3]. Exercise intolerance is therefore an important clinical feature in patients with COPD. The 6-minute walk test (6MWT) is a method of obtaining the 6-minute walk distance (6MWD) and is used to evaluate functional exercise capacity. Furthermore, the 6MWT is used to assess response to treatment and predicts morbidity and mortality in patients with COPD [4].

According to the European Respiratory Society/American Thoracic Society (ERS/ATS) guidelines, a flat corridor of at least 30 meters is required to perform a 6MWT [4, 5]. However, not all clinical facilities have such spaces. Therefore, treadmill walking tests offer advantages over overground walking tests, as limited space is needed, providing a safe environment without obstructions [5] and subjects do not have to turn, leading to an increase in walking distance [6].

To date, two studies have been conducted in assessing treadmill-based 6MWDs in patients with COPD. Both studies established a significantly greater mean overground 6MWD compared to a regular treadmill-based 6MWT (+102 and +51 m, respectively) [7, 8]. However, no difference was found between treadmill-based 6MWT and overground-based 6MWT in healthy subjects in three age groups (48–54 years: 25.1 m; 55–64 years: 15.2 m; 65–75 years: 11.2 m) [9].

Both COPD studies with the 6MWT have used regular fixed-paced treadmills. Conversely, the use of a self-paced treadmill, a feedback controlled function that adapts treadmill speed to its user, could be beneficial to adjust the walking speed more naturally and resulting in a more natural gait pattern compared to fixed speed treadmill walking [10]. In addition, the use of virtual reality during treadmill walking is becoming increasingly popular in the area of rehabilitation, since a virtual reality provides an engaging environment and induces a real life sensation [11]. The Gait Real-time Analysis Interactive Lab (GRAIL, Motekforce Link, Amsterdam, the Netherlands) system combines self-paced treadmill walking with virtual reality. Moreover, the GRAIL enables 3D motion capture to analyse gait patterns during walk tests. As the reproducibility of the self-paced treadmill-based 6MWT in patients with COPD remains currently unknown, it is necessary to assess the reproducibility of the 6MWT on the GRAIL and to compare the GRAIL 6MWT with the overground 6MWT. The aims of the current study were therefore to examine the reproducibility and validity of the 6MWT on the GRAIL in patients with COPD and healthy elderly.

## Materials & Methods

### Study design and sample

A cross-sectional observational study was conducted in CIRO, a centre of expertise for chronic organ failure located in Horn, the Netherlands. Sixty-one patients with COPD (FEV_1_/FVC <0.7) were recruited at pre-rehabilitation assessment between February 2014 and June 2015 [12]. Patients with walking aids, chronic oxygen use, orthopaedic ailments and/or neuromuscular co-morbidities affecting their walking patterns were excluded, as well as patients with a history of lung cancer, asthma, sarcoidosis, tuberculosis, and/or lung surgery. Forty-eight healthy elderly, aged 40–85 years, were recruited between July 2014 and October 2015. Healthy elderly were ineligible if respiratory or cardiac diseases, neuromuscular and/or orthopaedic ailments were present. The study complied with the Declaration of Helsinki and was approved by the Medical research Ethics Committees United (MEC-U) in the Netherlands (NL46880.060.13). Written consent was obtained from all participants.

### Assessment of 6MWD

**Fig 1:**
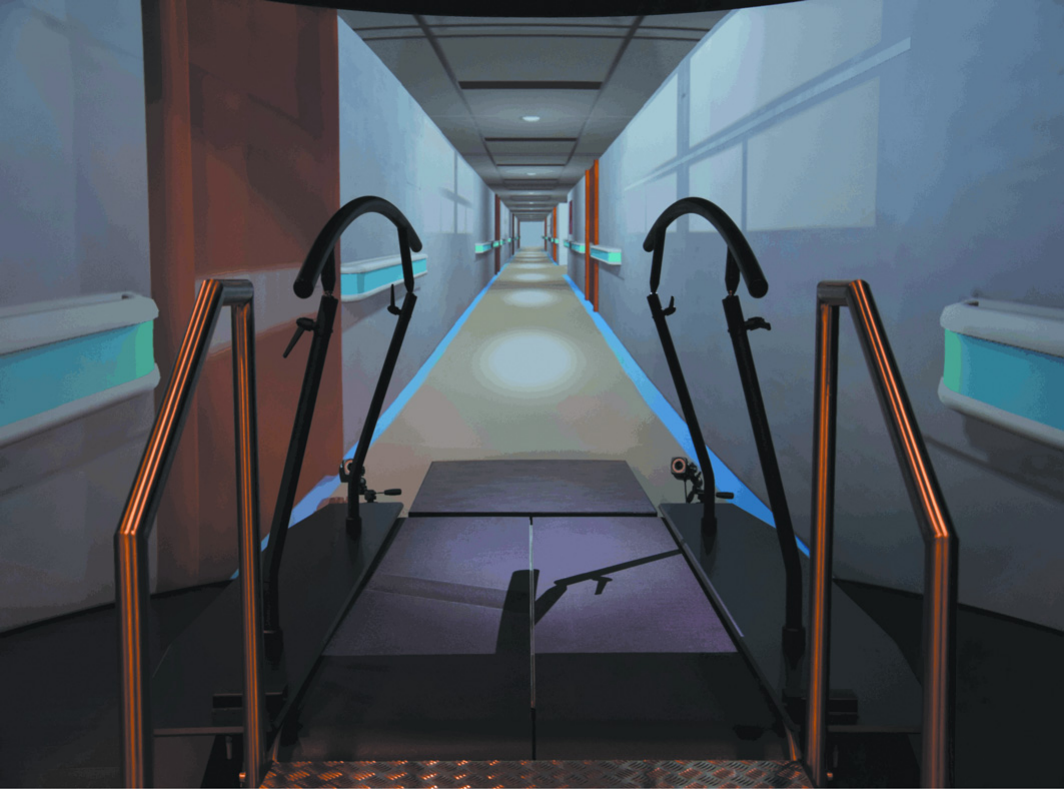
The 6MWT of the GRAIL system.

**Fig 2:**
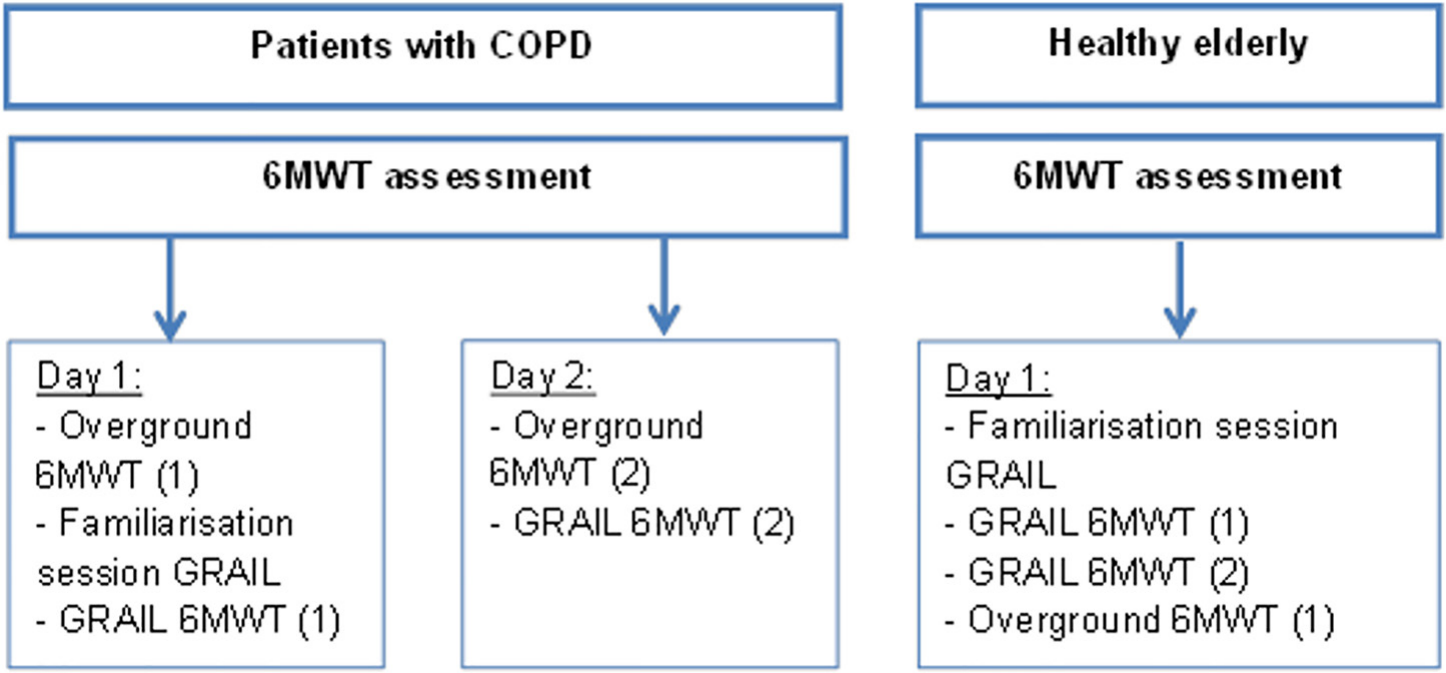
Protocol of 6MWT assessment.

The GRAIL (Motekforce Link, Amsterdam, the Netherlands) was used to assess the self-paced treadmill 6MWDs. The GRAIL comprises of a 3D motion analysis system with a dual-belt, instrumented treadmill and a virtual reality 180 degrees projection screen (Fig 1). Four retroflective surface markers were positioned on the anterior superior iliac spine and posterior superior iliac spine of the participant. Marker positions were detected using a ten camera VICON motion analysis system (100 Hz, Oxford Metrics Ltd., Oxford, UK) and automatically labelled in D-flow (Motekforce Link, Amsterdam, the Netherlands) in order to control treadmill speed via self-paced treadmill walking. The virtual hallway environment was synchronised with the treadmill speed. Participants were not allowed to hold onto the handrails and wore a safety harness during each GRAIL 6MWT. All participants performed one familiarisation session on the GRAIL (15–20 minutes) prior to the first GRAIL 6MWT. This session comprised of an explanation of the system and the use of the self-paced function of the treadmill. A four-minute familiarization walk on the treadmill was conducted as well in each participant. Therefore, participants could become accustomed to the virtual hallway environment and self-paced treadmill walking. After the familiarisation session, patients performed two GRAIL 6MWTs in two days during the pre-rehabilitation assessment and healthy adults performed the GRAIL 6MWTs in one day (Fig 2). One GRAIL session took 45 minutes. In addition, all participants performed an overground 6MWT in a 125 meter circular hallway, which took 15–20 minutes. Patients performed two overground 6MWTs during pre-rehabilitation assessment. Healthy elderly performed one overground 6MWT after the GRAIL 6MWTs with a resting period of at least 60 minutes. The overground 6MWD in healthy elderly was considered as the best overground walking distance. All 6MWTs were conducted according to the ERS/ATS guidelines [4]. The 6MWD and average walking speed were assessed. Walking speed was continuously assessed in D-flow and averaged over 6 minutes. Borg scores for both dyspnoea and fatigue were recorded before and after the 6MWT, as well as the heart rate and transcutaneous oxygen saturation using a pulse oximeter (Nonin, Care Fusion, San Diego, USA). During the GRAIL 6MWT, post heart rate and transcutaneous oxygen saturation were recorded after the subjects stepped down from the treadmill.

### Sample size calculation

Sample size calculation was based on the results of Stevens et al.[8]. Patients with lung diseases (76% COPD) achieved on average 374 ± 78 meters in the overground 6MWT and 323 ± 119 meters on a regular treadmill 6MWT. Using a posteriori sample size calculation with a power of 0.80, we calculated a sample size of 36 patients. We hypothesized that the difference in 6MWD will be smaller between overground and GRAIL walking in patients with COPD. We therefore included a larger number of subjects in both groups, which were available for this manuscript.

### Statistical analyses

The assumption of normally distributed data was checked with the Shapiro-Wilk test. If data were not normally distributed, non-parametric tests were used. Differences between overground 6MWTs in patients, differences between GRAIL 6MWTs in each group and differences between the best GRAIL 6MWT and best overground 6MWT in both groups were identified by paired sample *t*-tests or two related samples tests. Differences between groups were identified by independent *t*-tests or two independent samples tests. For consistency with previous studies, mean values of non-normally distributed variables are reported. Predicted values of the 6MWDs for patients and healthy elderly were calculated using the formula of Troosters et al. [13]. The Bland-Altman method was used to assess agreement between the two test conditions. The intra-class correlation coefficient (ICC) values between repetitive GRAIL 6MWTs and between the test conditions were calculated. All analyses were performed using the statistical package SPSS (version 22, IBM SPSS Statistics). Statistical significance was defined as a *p*-value <0.05.

## Results

In total 61 patients with COPD and 48 healthy elderly volunteered to participate. Patients had moderate to very severe COPD. Patients and healthy elderly subjects were comparable in age, height, weight and body mass index (BMI). The FEV_1_/FVC and FEV_1_% predicted differed between patients and healthy elderly (Table 1 and S1 Dataset).

**Table 1:** Sample characteristics.

	Patients with COPD	Healthy elderly
**Subjects n**	61	48
**Age years**	61.9 (± 6.8)	61.6 (± 6.1)
**Males %**	62.3	46.8
**Height m**	1.70 (± 0.09)	1.70 (± 0.09)
**Weight kg**	78.1 (± 17.7)	77.5 (± 12.8)
**BMI kg/m**^**2**^	27.0 (± 5.1)	26.6 (± 3.2)
**GOLD stage I-IV**	9/31/16/5	0
**FEV**_**1**_**/FVC %**	41.3 (± 10.6)	77.1 (± 4.5)*
**FEV**_**1**_**, % predicted**	57.6 (± 20.0)	119.1 (± 16.4)* ^a^
**Non smoker**	1	18
**Former smoker**	56	28
**Current smoker**	4	2

Data are presented as n or mean ± standard deviation. BMI: body mass index; FEV_1_: forced expiratory volume in 1s; FVC: forced vital capacity; Non smoker: < 1 pack year; Former smoker: quitted smoking before participation at this study with ≥ 1 pack years; Current smoker: smoked during participation at this study ≥1 pack years.

*: significant difference between patients and healthy elderly (p<0.05).

^a^: nonparametric test have been used.

### Overground 6MWT

In the overground condition, patients walked 481.4 m (95% CI 465.7–497.0 m) in the first 6MWT and 506.2 m (95% CI 488.3–524.0 m) in the second 6MWT, with an average increase of 24.8 m (95% CI 15.2–34.4 m; p<0.001). Eighty percent of patients improved during the second overground 6MWT. The best 6MWD in patients was 511.0 m (95% CI 494.5–527.6 m), which was 79% (95% CI 76–82%) of the predicted value. At the end of the best overground 6MWT, patients had Borg dyspnoea and fatigue scores of 5.4 (95% CI 4.8–6.0) and 5.3 (95% CI 4.7–5.9), respectively. Forty-nine percent of patients desaturated (SpO_2_nadir ≤ 88% [14]) during their best overground test. Oxygen saturation decreased by 7.1% (95% CI 5.6–8.6%) and heart rate increased by 29.5 bpm (95% CI 26.4–32.5 bpm).

Healthy elderly covered 668.8 m (95% CI 647.4–690.1 m), which was 103% (95% CI 100–106%) of the predicted value. At the overground 6MWT, healthy elderly had Borg dyspnoea and fatigue scores of 1.2 (95% CI 0.9–1.5) and 1.2 (95% CI 0.9–1.5), respectively. Oxygen saturation decreased by 1.2% (95% CI 0.2–2.2%) and heart rate increased by 47.3 bpm (95% CI 42.8–51.9 bpm). Difference in best overground 6MWD of patients and healthy elderly was 157.8 m (95% CI 131.5–184.0 m; p<0.001). All overground 6MWT outcomes are documented in S1 Dataset.

### GRAIL-based 6MWT

Patients walked 449.7 m (95% CI 426.8–472.5 m) in the first GRAIL 6MWT and 476.4 m (95% CI 454.2–498.7 m) in the second test (Table 2), with an average increase of 26.8 m (95% CI 13.9–39.6 m; p<0.001). Seventy-seven percent of all patients performed the second GRAIL test during the pre-rehabilitation assessment. The ICC value of the reproducibility of the GRAIL 6MWTs was 0.80 (95% CI 0.61–0.89) and 75% of patients improved during the second GRAIL test. The best GRAIL 6MWD in patients was 483.7 m (95% CI 462.1–505.3 m) (Table 3). At the end of the best GRAIL 6MWT, patients had Borg dyspnoea and fatigue scores of 4.8 (95% CI 4.2–5.4) and 4.6 (95% CI 4.0–5.2), respectively. Oxygen saturation decreased by 2.0% (95% CI 0.9–3.13%) and heart rate increased by 19.1 bpm (95% CI 16.4–21.8 bpm). Nine patients (14.8%) desaturated during their best GRAIL 6MWT.

**Table 2:** The GRAIL 6MWTs.

			GRAIL 6MWT (1)		GRAIL 6MWT (2)	
			Patients	Healthy elderly	Patients	Healthy elderly
**6MWD, m**			449.7 ± 89.3	639.9 ± 77.1^#^	476.4 ± 86.8 ^a^ *	689.5 ± 62.2* ^#^
**Walking speed, km/h**			4.5 ± 0.9	6.4 ± 0.8^#^	4.8 ± 0.9 ^a^ *	6.9 ± 0.7*^#^
**Oxygen saturation, %**	Pre		94.9 ± 1.8 ^a^ ^b^	97.2 ± 1.2 ^a^ ^#^	95.5 ± 1.6 ^a^ *	97.2 ± 1.0 ^a^ ^#^
	Post		93.4 ± 4.4 ^a^	97.4 ± 0.9 ^a^ ^#^	93.2 ± 4.8 ^a^	97.2 ± 1.3 ^a^ ^#^
**Δ Post-Pre Oxygen saturation, %**			-1.5 ± 3.9 ^a^ ^b^ ^ǂ^	0.2 ± 1.0 ^a^ ^ǂ^ ^#^	-2.3 ± 4.3 ^ǂ^ *	0.0 ± 1.0 ^a^ ^#^
**Heart rate, bpm**	Pre		82.4 ± 14.9 ^b^	65.0 ± 11.1^#^	83.7 ± 14.7	68.0 ± 12.2*^#^
	Post		100.9 ± 16.4	89.3 ± 21.6^#^	102.5 ± 17.9	99.5 ± 21.1 ^a^ *
**Δ Post-Pre Heart rate, bpm**			18.7 ± 11.0 ^a^ ^b^ ^ǂ^	24.3 ± 15.5^ǂ^	18.8 ± 10.0 ^a^ ^ǂ^	31.5 ± 14.2 ^ǂ^ * ^#^
**Borg dyspnoea, points**	Pre		1.3 ± 1.1 ^a^	0.2 ± 0.4 ^a^ ^#^	1.4 ± 1.2 ^a^	0.3 ± 0.5 ^a^ ^#^
	Post		4.5 ± 2.0 ^a^	1.1 ± 1.0 ^a^ ^#^	4.6 ± 2.3 ^a^	1.2 ± 1.1 ^a^ ^#^
**Δ Post-Pre Borg dyspnoea, points**			3.2 ± 2.0 ^ǂ^	0.9 ± 1.0 ^a^ ^ǂ^ ^#^	3.2 ± 2.2 ^ǂ^	0.9 ± 0.9 ^a^ ^ǂ^ ^#^
**Borg fatigue, points**	Pre		1.5 ± 1.3 ^a^	0.3 ± 0.6 ^a^ ^#^	1.5 ± 1.3 ^a^	0.3 ± 0.6 ^a^ ^#^
	post		4.3 ± 2.4 ^a^	1.3 ± 1.2 ^a^ ^#^	4.6 ± 2.3 ^a^	1.3 ± 1.1 ^a^ ^#^
**Δ Post-Pre Borg fatigue, points**			2.8 ± 2.3 ^ǂ^	1.0 ± 1.1 ^a^ ^ǂ^ ^#^	3.1 ± 2.1 ^ǂ^	1.0 ± 1.1 ^a^ ^ǂ^ ^#^

Data are presented as n or mean ± standard deviation. Bpm: beats per minute.

^ǂ^: significant difference between pre and post symptoms per trial.

*: significant difference between the first and second GRAIL 6MWT within each group.

^#^: significant difference between patients and healthy elderly.

^a^: nonparametric test have been used.

^b^: missing value in patients, N = 60.

**Table 3:** Best 6MWT in the overground and GRAIL condition.

		Overground 6MWT		GRAIL 6MWT		Δ Conditions in patients, p-value	Δ Conditions in HMO, p-value
		Patients	Healthy elderly	Patients	Healthy elderly		
**6MWD, m**		511.0 ± 64.6	668.8 ± 73.6^#^	483.7 ± 84.5 ^a^	692.3 ± 62.0^#^	<0.001 ^a^	<0.001
**Walking speed, km/h**		5.1 ± 0.6	6.7 ± 0.7^#^	4.8 ± 0.8 ^a^	6.9 ± 0.7^#^	<0.001 ^a^	<0.001
**Oxygen saturation, %**	Pre	94.9 ± 2.1 ^a^	96.6 ± 1.4 ^a^ ^#^	95.2 ± 1.5 ^a^	97.2 ± 1.0 ^a^ ^#^	0.36 ^a^	0.01 ^a^
	Post	87.8 ± 6.7 ^a^	94.5 ± 3.4 ^a^ ^#^	93.1 ± 4.7 ^a^	97.2 ± 1.2 ^a^ ^#^	<0.001 ^a^	<0.001 ^a^
**Δ Post-Pre Oxygen saturation, %**		-7.1 ± 5.9 ^a^ ^ǂ^	-1.2 ± 3.4 ^a^ ^#^	-2.0 ± 4.4 ^a^ ^ǂ^	0.0 ± 0.9 ^a^ ^#^	<0.001 ^a^	0.02 ^a^
**Heart rate, bpm**	Pre	85.0 ± 13.6	72.3 ± 11.9^#^	83.5 ± 14.5	67.1 ± 11.9^#^	0.30	0.001
	Post	114.5 ± 15.8	119.6 ± 18.6	102.7 ± 18.3	99.7 ± 21.1	<0.001	<0.001
**Δ Post-Pre Heart rate, bpm**		29.5 ± 11.8 ^a^ ^ǂ^	47.3 ± 15.7 ^a^ ^#^	19.1 ± 10.5^ǂ^	32.6 ± 15.1^ǂ^ ^#^	<0.001 ^a^	<0.001 ^a^
**Borg dyspnoea, points**	Pre	1.4 ± 1.2 ^a^	0.1 ± 0.3 ^a^ ^#^	1.3 ± 1.3 ^a^	0.2 ± 0.3 ^a^ ^#^	0.51 ^a^	0.02 ^a^
	Post	5.4 ± 2.2 ^a^	1.2 ± 1.0 ^a^ ^#^	4.8 ± 2.3 ^a^	1.2 ± 1.0 ^a^ ^#^	0.01 ^a^	0.95 ^a^
**Δ Post-Pre Borg dyspnoea, points**		4.0 ± 2.3 ^a^ ^ǂ^	1.1 ± 0.9 ^a^ ^#^	3.4 ± 2.2 ^ǂ^	1.0 ± 0.9 ^a^ ^ǂ^ ^#^	0.010 ^a^	0.54 ^a^
**Borg fatigue, points**	Pre	1.5 ± 1.3 ^a^	0.2 ± 0.3 ^a^ ^#^	1.5 ± 1.4 ^a^	0.3 ± 0.6 ^a^ ^#^	0.67 ^a^	0.12 ^a^
	post	5.3 ± 2.3 ^a^	1.2 ± 1.1 ^a^ ^#^	4.6 ± 2.4 ^a^	1.3 ± 1.1 ^a^ ^#^	0.01 ^a^	0.57 ^a^
**Δ Post-Pre Borg fatigue, points**		3.7 ± 2.2 ^a^ ^ǂ^	1.1 ± 1.0 ^a^ ^ǂ^ ^#^	3.2 ± 2.1 ^ǂ^	1.1 ± 1.0 ^a^ ^ǂ^ ^#^	0.02 ^a^	0.77 ^a^

Data are presented as n or mean ± standard deviation. Bpm: beats per minute.

^ǂ^: significant difference between pre and post symptoms per trial.

^#^: significant difference between patients and healthy elderly.

^a^: non parametric tests have been used.

Healthy elderly walked 639.8 m (95% CI 617.5–662.3 m) at first, and then 689.5 m (95% CI 671.4–707.6 m) (Table 2), with an average increase of 49.6 m (95% CI 37.0–62.3 m, p<0.001). Ninety-four percent of all healthy elderly subjects performed all GRAIL test in one day. The ICC value of the reproducibility of the GRAIL 6MWTs was 0.65 (95% CI 0.05–0.86) and 90% of healthy elderly improved during the second GRAIL 6MWT. The best 6MWD was 692.3 m (95% CI 674.3–710.3 m) (Table 3). At the end of the best GRAIL 6MWT, healthy elderly had Borg dyspnoea and fatigue scores of 1.2 (95% CI 0.9–1.5) and 1.3 (95% CI 1.0–1.7), respectively. Oxygen saturation did not change (0.0%; 95% CI 0.2–0.3%; p = 0.67), whereas heart rate increased by 32.6 bpm (95% CI 28.2–37.0 bpm). All GRAIL 6MWT outcomes are documented in S1 Dataset.

### GRAIL versus overground 6MWT

**Fig 3:**
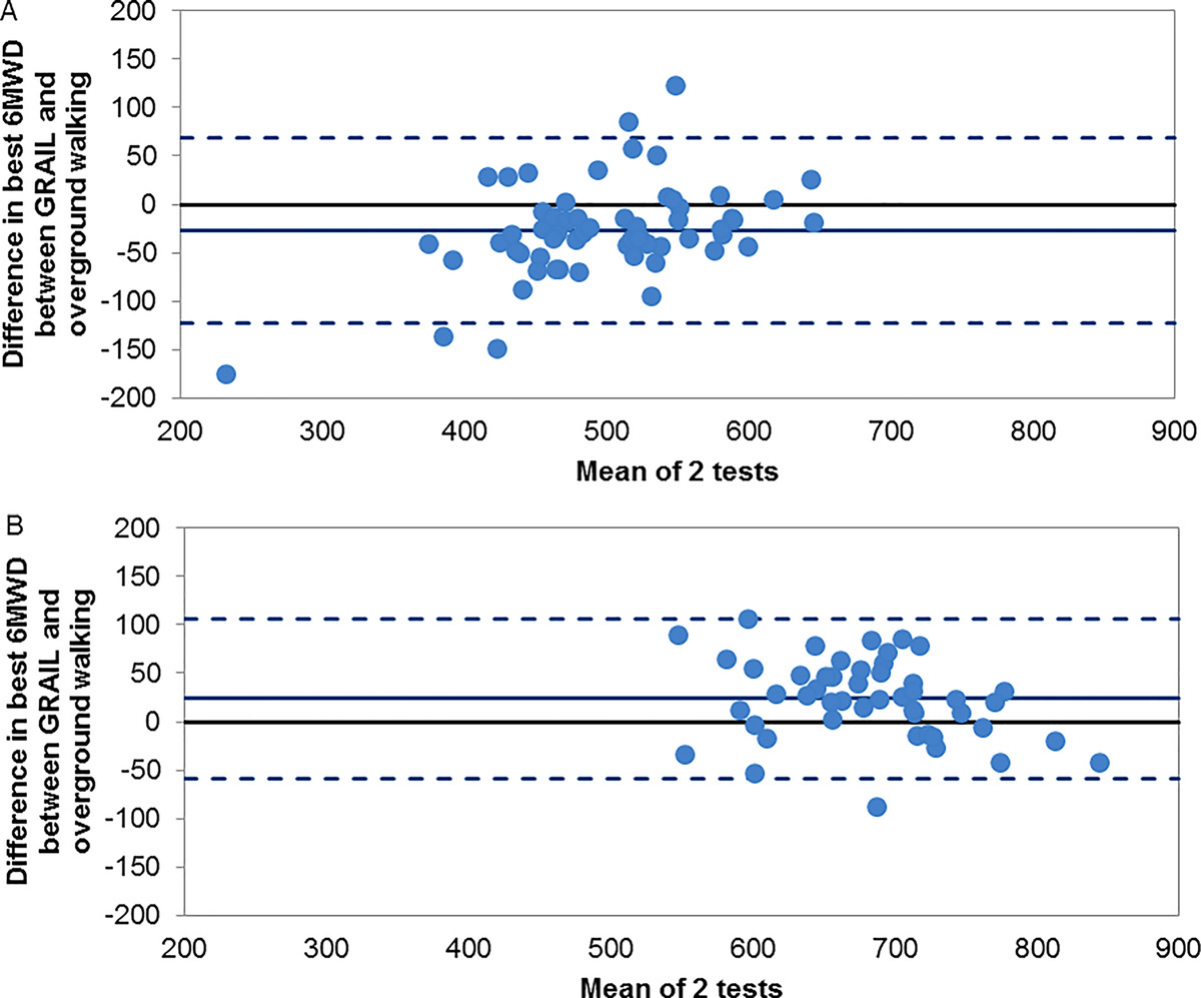
**Differences between the best overground 6MWT and GRAIL 6MWT in (3A) patients with COPD and (3B) healthy elderly.** Bland-Altman plots of the difference between the best overground 6MWT and best GRAIL 6MWT, plotted against the mean value of the two tests in each group. The central solid lines represent the mean difference between the two methods, whereas the lower and upper dashed lines represent the limits of agreement (1.96 SD of mean value).

On average, the best GRAIL-based 6MWD in patients was significantly shorter than the best overground 6MWD (-27.3 ± 49.0 m, p<0.001). Conversely, the GRAIL 6MWD in healthy elderly was significantly greater than the overground 6MWD (23.6 ± 41.4 m, p<0.001; Table 3). The Bland-Altman plot (Fig 3) confirms that the majority of patients walked further overground compared to the GRAIL, while the majority of the healthy elderly walked further in the GRAIL condition. Validity of the GRAIL 6MWT compared to overground 6MWT was assessed in both groups, with ICC values of 0.74 (95% CI 0.51–0.86) for patients and 0.77 (95% CI 0.53–0.88) for healthy elderly.

## Discussion

The present study provides the first evaluation of the reproducibility and validity of the 6MWT assessed by the GRAIL in patients with COPD. It extends previous work on treadmill based 6MWTs by assessing the 6MWD using virtual reality and self-paced treadmill walking. On average, patients increased their 6MWD between the first and second walk test equally in the overground and GRAIL condition. Furthermore, the 6MWT on the GRAIL showed good reproducibility with ICC values of 0.65 for healthy elderly and 0.80 for patients. The best 6MWD in patients was obtained in overground walking, while healthy elderly covered greater distances using the GRAIL. Moreover, the 6MWT on the GRAIL showed to have good construct validity with ICC values of 0.74 and 0.77. Therefore, these results indicate that the 6MWD could be reliably and validly assessed by the GRAIL in patients with COPD and healthy elderly. The 6MWD between the first and second trial improved equally in the overground and GRAIL 6MWT in patients with COPD. These results are similar to existing literature in assessing the 6MWD within this patient group [15, 16]. Larger increases in the second GRAIL 6MWT were found in healthy elderly compared to patients, despite all participants undergoing one familiarisation session prior to the first GRAIL 6MWT. A possible explanation is that treadmill walking requires different energy expenditure in each of the subject groups [7]. Another possibility could be that self-paced treadmill walking required more effort of the patient than healthy elderly, due to muscle weakness, balance deficits and/or anxiety [17–19]. In addition, adaptability towards learning new tasks might be affected in patients with COPD, as declines in a number of cognitive functions have been observed previously [20, 21].

Patients achieved on average a lower 6MWD (-27.5 m) on the GRAIL compared to overground walking. This difference in 6MWD between the test conditions is smaller than previous studies using regular treadmills have established [7, 8]. Our findings are supported by less increase in the Borg dyspnoea and fatigue scores, less decrease in oxygen saturation and less increase in heart rate in the GRAIL condition. Healthy elderly, however, achieved greater 6MWDs while walking on the GRAIL, which is in contrast with the findings of Elazzazi et al. [9]. Healthy elderly did not differ in their degree of dyspnoea or fatigue between their best overground and best GRAIL test. Therefore, we can assume that healthy elderly experienced equal exertion in performing the 6MWT in each condition. However, this was not seen in the heart rate, as the heart rates were higher after the overground 6MWT compared to the GRAIL 6MWT. Our study showed smaller differences in 6MWD between the test conditions, which might be due to the use of the self-paced treadmill walking. Self-paced treadmill walking offers a more naturally adjustment of walking speed, which could lead to a more natural gait pattern compared to fixed speed treadmill walking [10]. In addition, the overground track in this study required multiple turnarounds compared to the GRAIL condition. However, the turns were larger than most clinical settings use (30 meters). The virtual reality environment could have created a more realistic environment by providing optic flow. By combining the self-paced treadmill walking and virtual reality, a greater 6MWD might have been achieved compared to regular treadmill walking [11, 22].

Despite the fact that the familiarisation session is only performed prior to the first GRAIL 6MWT, which could have led to less distance covered in the second GRAIL 6MWT, our results indicate that 75% of patients improved their walk distance during the second GRAIL test. This is comparable to the 80% of patients who improved during their second overground test. Therefore, we consider this effect to be minimal. Concerning the duration of a GRAIL 6MWT. One GRAIL session takes more time to perform compared to the overground 6MWT. In addition, the GRAIL is less accessible to all centres compared to regular treadmills. We do however not argue that the GRAIL should be implemented everywhere to assess the 6MWD only. The GRAIL is a unique method to conduct analysis of gait impairments in patients with COPD, as these patients have reported walking as one of the most problematic activity in daily life [23]. Future study should therefore focus on gait assessment in patients with COPD. Furthermore, predicted distance values and minimal clinical important difference (MCID) of the 6MWT for patients with COPD are available in the overground condition. As these have not been determined in the GRAIL condition, next steps should be to derive new reference values for the GRAIL condition in healthy elderly subjects and to determine MCID, if the GRAIL will be used for further assessment of patients with COPD.

A first limitation of this study is that healthy elderly performed one overground 6MWT, which might have led to a shorter best overground 6MWD. However, a previous study found a minimal difference of 5 meters between two overground 6MWTs in healthy elderly [24]. Therefore, healthy elderly could achieve their best 6MWD during their first overground 6MWT. The second limitation is that there is a time gap between the GRAIL 6MWT and the post assessment of oxygen saturation levels and heart rates compared to the overground 6MWT. It is possible that conclusions based on the differences in heart rates and oxygen saturation might not explain the differences between the test conditions. A third limitation is that GOLD stage 4 patients are less represented in this study. Therefore, our findings should be carefully interpreted for stage 4 patients. Moreover, complex patients with COPD were excluded, as this is the first study to assess the 6MWT using the GRAIL. Consequently, patients should be able to perform the GRAIL tests without using the handrails and be able to control the self-paced treadmill. A fourth limitation is that this study is a monocentric study, as access to the GRAIL in other centres is currently limited. Moreover, CIRO is a specialized pulmonary rehabilitation clinic, which may limit the external validity of current findings. A fifth limitation is that the learning effect of 6MWT on the GRAIL in repetitive tests of more than two trials has not been established. Consequently, it is not known if the learning effect attenuates in a third GRAIL 6MWT. Another limitation is that balance during treadmill walking might be affected. However, the GRAIL provides the opportunity to assess quality of gait (e.g. balance) continuously in a virtual reality environment and during self-paced walking, which is not possible using a regular treadmill or in an overground condition. As a result, new insights in determinants of walking balance in patients with COPD could be achieved by using this system.

In conclusion, the GRAIL is a promising system to assess the 6MWD in patients with COPD and healthy elderly. The 6MWD assessed by the GRAIL seems to be more comparable to the 6MWDs assessed overground than previous studies on treadmills have reported. Furthermore, good construct validity and reproducibility were established in assessing the 6MWD using the GRAIL in patients with COPD and healthy elderly.

## Supporting Information

S1 Dataset Subjects’ characteristics and 6MWT outcomes. GOLD I defined as FEV_1_ ≥ 80% predicted. GOLD II defined as FEV_1_ 50% ≤ FEV_1_ < 80% predicted. GOLD III defined as 30% ≤ FEV_1_ < 50% predicted. GOLD IV defined as FEV_1_ < 30% predicted.
(XLSX)
